# A Hot-spot of In-frame Duplications Activates the Oncoprotein AKT1 in Juvenile Granulosa Cell Tumors

**DOI:** 10.1016/j.ebiom.2015.03.002

**Published:** 2015-03-06

**Authors:** Laurianne Bessière, Anne-Laure Todeschini, Aurélie Auguste, Sabine Sarnacki, Delphine Flatters, Bérangère Legois, Charles Sultan, Nicolas Kalfa, Louise Galmiche, Reiner A. Veitia

**Affiliations:** a Institut Jacques Monod, Université Paris Diderot, CNRS UMR7592, Paris 75013, France; b Université Paris Diderot-Paris VII, 75205 Paris Cedex 13, France; c Hôpital Necker Enfants-Malades, Paris, France; d Université Paris Descartes-Paris V, 75015 Paris, France; e Molecules Thérapeutiques in silico, Université Paris Diderot, INSERM UMR973, Paris 75013, France; f Deparment of Pediatric Endocrinology, University Hospital of Montpellier, Montpellier, France; g Deparment of Pediatric Surgery, Hôpital Lapeyronie, CHU Montpellier, France

**Keywords:** AKT1, Ovary, Juvenile granulosa cell tumor, Activating mutation, Oncogene, Oncoprotein

## Abstract

**Background:**

Ovarian granulosa cell tumors are the most common sex-cord stromal tumors and have juvenile (JGCTs) and adult forms. In a previous study we reported the occurrence of activating somatic mutations of *Gαs*, which transduces mitogenic signals, in 30% of the analyzed JGCTs.

**Methods:**

We have searched for alterations in other proteins involved in ovarian mitogenic signaling. We focused on the PI3K–AKT axis. As we found mutations in AKT1, we analyzed the subcellular localization of the mutated proteins and performed functional explorations using Western-blot and luciferase assays.

**Findings:**

We detected in-frame duplications affecting the pleckstrin-homology domain of AKT1 in more than 60% of the tumors occurring in girls under 15 years of age. The somatic status of the mutations was confirmed when peritumoral DNA was available. The JGCTs without duplications carried point mutations affecting highly conserved residues. Several of these substitutions were somatic lesions. The mutated proteins carrying the duplications had a non-wild-type subcellular distribution, with a marked enrichment at the plasma membrane. This led to a striking degree of AKT1 activation demonstrated by a strong phosphorylation level and by reporter assays.

**Interpretation:**

Our study incriminates somatic mutations of *AKT1* as a major event in the pathogenesis of JGCTs. The existence of AKT inhibitors currently tested in clinical trials opens new perspectives for targeted therapies for these tumors, which are currently treated with standard non-specific chemotherapy protocols.

## Highlights

• We detected duplications affecting the pleckstrin-homology domain of AKT1 in > 60% of the Juvenile Granulosa cell tumors studied.
• The JGCTs without duplications carried point mutations affecting highly conserved residues.
• Mutated proteins with duplications displayed an enrichment at the plasma membrane and a striking phosphorylation level.

## Graphical abstract

**Figure:**
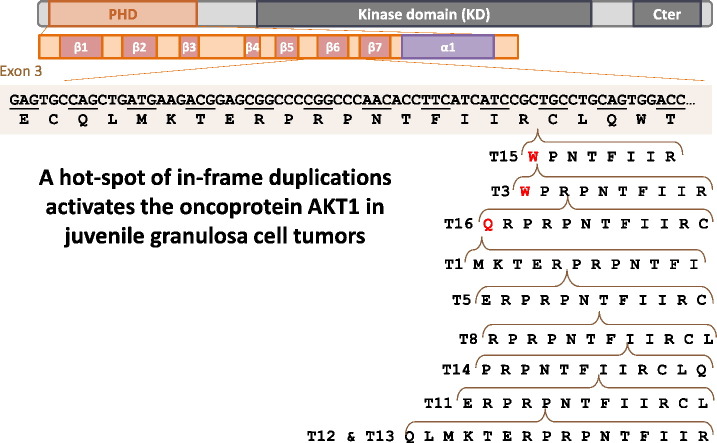

## 1 Introduction

Sex cord stromal tumors involve granulosa, theca or stromal cells, alone or combined. The most common ones are ovarian granulosa cell tumors (GCTs), which represent up to 5–8% of all ovarian tumors (Pectasides et al., 2008, Young and Scully, 1992). Two distinct subtypes have been described based on clinical presentation and histology: the juvenile and the adult forms. Five percent of GCTs occur in the prepubertal period and are often uncovered by a precocious pseudo-puberty and/or dysmenorrhea (Fleming et al., 2010). Although advanced-stage disease can be observed, most of the juvenile GCTs (JGCTs) are detectable at an early stage but recurrence and metastases are possible (Kalfa et al., 2007). The adult form (AGCTs) most commonly appears during the perimenopausal period. AGCTs are characterized by a tendency to late recurrence (Pectasides et al., 2008). The pathophysiological mechanisms underlying GCTs are still unclear. However, a recurring somatic mutation has been identified in the sequence of *FOXL2*, which encodes a transcription factor, in more than 95% of AGCTs (Shah et al., 2009). This mutation perturbs TGF-beta signaling in granulosa cells (Rosario et al., 2012). With regard to JGCTs, in a previous study we showed that FOXL2 expression was absent or reduced in the granulosa cells of a number of patients. Interestingly, the patients with absent/reduced FOXL2 expression displayed higher mitotic activity in the tumor and more advanced oncological stages. Furthermore, all recurring tumors displayed extinction of FOXL2 (Kalfa et al., 2007). In another study, we reported the occurrence of the activating somatic mutations R201C and R201H of *Gαs* in one third of the analyzed JGCTs. This mutation may lead to a constitutive activation of mitogenic FSHR signaling in the latter (Kalfa et al., 2006). FSHR signaling also activates the oncoprotein AKT (Hunzicker-Dunn et al., 2012) that phosphorylates and inhibits transcription factors such as those belonging to the Forkhead box protein O family (FOXO) (Telikicherla et al., 2011). Because not only FSH but other mitogens such as IGFs also signal through PIK3CA and AKT in granulosa cells (Hunzicker-Dunn et al., 2012, Baumgarten et al., 2014, Reynaud et al., 2010), we hypothesized that alterations of this pathway might be involved in the molecular etiology of JGCTs. Thus, we searched for mutations in the genes encoding these proteins in a cohort of 16 tumors occurring in girls under 15 years of age. Recently, mutations in *PIK3CA*, which encodes the catalytic subunit of PI3K, were identified in various human cancers. Since 80% of these mutations cluster within exons 9, 18 and 20, we focused on them and found no alterations. With regard to AKT, we show here that more than 60% (i.e. 10/16) of the JGCT samples harbor in-frame duplications that affect the pleckstrin-homology domain (PHD) and activate AKT1 and that the rest of the tumors often display several potentially damaging point mutations. Our study points to AKT1 as a major driver in the pathogenesis of JGCTs.

## 2 Materials and Methods

### 2.1 Patients

This study involves a cohort of 16 histologically proven JGCTs, occurring in girls under 15 years of age, collected between 1994 and 2014 (from the Necker-Enfants Malades Hospital, Paris and the University Hospital Montpellier tumor repositories). The available clinical data are displayed in Table 1. This study was validated by the Ethics Committees of the two tumor repositories that contributed the samples.

### 2.2 Nucleic Acid Extraction and Sequencing

Twelve samples were formalin-fixed paraffin-embedded (FFPE) (T1–T12). Four tumors were obtained as frozen samples (T13–T16). We isolated genomic DNA and RNA from FFPE tumors using the AllPrep DNA/RNA FFPE Kit (Qiagen) and the frozen ones were processed using standard procedures. To assess the somatic status of the mutations, we extracted DNA from manually isolated peritumoral tissue fragments (only available for 8 samples). We also analyzed the COV434 cell line, supposed to derive from a JGCT (Van den Berg-Bakker et al., 1993).

Sequencing of exon 8 of the *Gαs* gene, potentially harboring the activating mutations R201C and R201H, was performed as described (Kalfa et al., 2006). Exons 9, 18 and 20 of the *PI3KCA* (Li et al., 2005) and all of the exons of *AKT1* were amplified from gDNA using the primers described below. For nested/semi-nested PCRs, when required, we used the primers F2 and/or R2 from the list below. Sanger sequencing was performed by MWG-Biotech-AG according to their in-house procedures.

Exon1-F1: 5′TGGCCTCACATTCAGCTTCCTT3′, Exon1-F2: 5′AGCGCCAGCCTGAGAGGA3′,
Exon1-R1: 5′AGGGCACAGGCACTCACAGA3′
Exon2-F1: 5′TGTCCTGGCACACCCAGTT3′, Exon2-F2: 5′AGGGTCTGACGGGTAGAGT3′,
Exon2-R1: 5′GCAAAGAGGGCTCCAGCCAA3′
Exon3-F1: 5′ATGCACGCAGACAGAGGCT3′, Exon3-F2: 5′ATCCCCGTGTCCCTCCTAAGC3′,
Exon3-R1: 5′GAGGATGGCTACAGGCAGAGGT3′
Exon4-F1: 5′TGTGGAACCACGCTTGTGA3′, Exon4-F2: 5′TGAAAGACGTGGGGTGGAGC3′,
Exon4-R1: 5′CCTCCACAGTCCAAGGCA3′, Exon4-R2: 5′CAGGCACAGGCAGAAGTGG3′
Exon5-F1: 5′TTGCTGACCCTGGTGCCTG3′, Exon5-R1: 5′AGGAAGGGGTGCCTGGAGT3′,
Exon5-R2: 5′CACCCCGCACCCTCATCT3′
Exon6-F1: 5′AAGGAAGTCATCGTGGCCAAGGT3′, Exon6-R1: 5′TAAAGCCCTCACGTGCCCAAGAA3′,
Exon6-R2: 5′AGCTCACCCAGCCCTGCTTTACA3′
Exon7-F1: 5′TCAGGCGACGTGGTATCAAGC3′, Exon7-R1: 5′CCCTAACTCAGCAGGAACAAGTCA3′,
Exon7-R2: 5′ACAGGCCGCGAAGTCCATC3′
Exon8-F1: 5′CACGGCTGTGCCTCAGGTT3′, Exon8-R1: 5′GTGATCTTAATGTGCCCGTCCTTG3′,
Exon8-R2: 5′CTCAGGTCAGTGCCGCCA3′
Exon9-F1: 5′ACTGACCTGAGGCCACCTTT3′, Exon9-R1: 5′AGCATTGCGTGTGCTCAGGA3′,
Exon9-R2: 5′GACGCAGCAACGCGTATG3′
Exon10-F1: 5′GCCGAGTCCTGCCCATCT3′, Exon10-F2: 5′AGGTGCTGGAGGACAATGACTA3′,
Exon10-R1: 5′GGATGAGGGGATGGAGGTGTA3′
Exon11-F1: 5′CGACACTGTGGCCTTGTTTCCT3′, Exon11-R1: 5′CGTGCATGCGTGAGTGTGGATA3′,
Exon11-R2: 5′ATGCGTGCGCGTGAATATGC3′
Exon12-F1: 5′AAGCTCATGACTGTCCCGTCTG3′, Exon12-R1: 5′ACTGCCTCCCACCCTGATCATT3′,
Exon12-R2: 5′CTCTCTGAGTGTGGAGAGAAAAGG3′
Exon13-F1: 5′GTTGGCTTCCTACTGGAGCTGT3′, Exon13-F2: 5′TGGAGGTGGCAGGGAGGT3′,
Exon13-R1: 5′CCTCTCCATCCCTCCAAGCTAT3′ Exon13-R2: GTTGGCTTCCTACTGGAGCTGT3′.

**Fig. 1:**
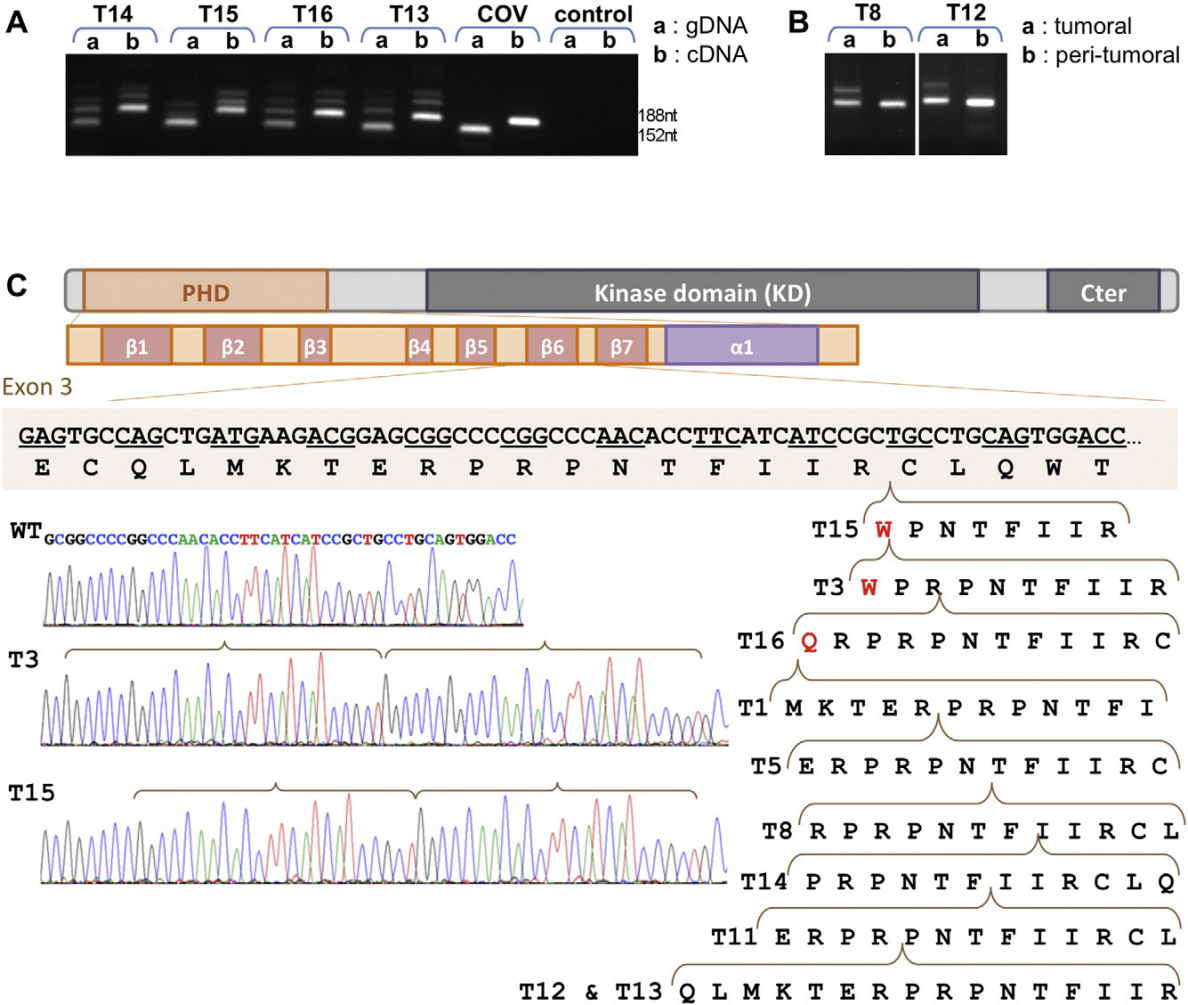
Tandem duplications within exon 3 of *AKT1* in JGCTs. A: Agarose gels showing the migration of the amplicons of exon 3 of *AKT1* from gDNA (a) and cDNA (b) in the 4 tumors of the JGCT cohort and in the COV434 JGCT-derived cell line. B: Electrophoretic migration of the amplicons involving exon 3 from gDNA of two tumors T8 and T12 (a) and from the corresponding peritumoral gDNA (b). The absence of the longer bands in the latter confirms the somatic nature of the duplications. C: Simplified representation of the AKT1 protein and of the PHD with its secondary structural features (beta strands from 1–7 and the C-terminal helix, according to crystallographic data from the Protein Data Bank). The sequences of 9 different duplications are displayed, along with two examples of Sanger sequencing traces. The tandemly duplicated sequences are highlighted by horizontal brackets pointing to the position of the insertion in the wild-type sequence. The duplications involve totally or partially the 6th beta strand of the PHD. Three of the duplications break a codon, which leads to a change of one residue (in red). The official names (according to https://www.mutalyzer.nl/name-checker) and details of the duplications are provided in the Supplementary material.

The primers used to obtain the amplicons shown in Fig. 1A and B were

gDNA-F1: 5′ATGCACGCAGACAGAGGCT3′, gDNA-R1: 5′CACGTACCGCTCCTCAGGAGT3′
cDNA-F2: 5′GCAGGATGTGGACCAACGTGA3′, cDNA-R2: 5′TCTGGATGGCGGTTGTCCACT3′.

### 2.3 Cell Culture

HeLA cells were used for protein localization and functional studies. They were grown in DMEM-F12 medium (Gibco®, Life Technologies, Grand Island, NY, USA), supplemented with 10% fetal bovine serum (FBS) and 1% penicillin/streptomycin (Invitrogen-Gibco, Life Technologies, Grand Island, NY, USA).

### 2.4 AKT1 Expression Constructs

The plasmids driving the expression of wild-type and mutated AKT1 fused to the mCherry protein were constructed by fusion PCR. Briefly, for the insertion mutations, two PCRs were performed to generate the 5′ and 3′ portions of the AKT1 coding sequence using, respectively, AKT1RED–EcoR1-F primer and the corresponding mutagenic R primer and AKT1-F2 primer along with AKT1RED–BamH1-R. After purification of the PCR products, they were quantified, mixed in similar amounts and allowed to undergo eight cycles of PCR in the absence of primers, to generate the full-length mutated coding regions. Then, a final PCR reaction was performed using the EcoR1–BamHI primers. For E17K, we used the primers E17K F and AKT1RED–BamH1-R to generate the amplicon in a single PCR. The amplified EcoR1–BamHIs were cloned (EcoR1–BamHI) into digested pDsRed vector to produce fusion proteins in frame with the mCherry. All constructs were sequenced to exclude the presence of PCR-induced mutations. The sequences of the primers used are the following:

AKT1RED–EcoR1-F: 5′AGCTTCGAATTCGCCACCATGAGCGACGTGGCTATTGTGAAGG3′
AKT1-F2: GTGGACCACTGTCATCG3′
AKT1RED–BamH1-R: 5′ACCGGTGGATCCCG GGCCGTGCCGCTGGCCGAGTAGGAGAAC3′
InsertionT3R: 5′CGATGACAGTGGTCCACTGCAGGCAGCGGATGATGAAGGTGTTGGGCCGGGGCCAGCGGATGATGAAGG3′
InsertionT8R: 5′CGATGACAGTGGTCCACTGCAGGCAGCGGATGATGAAGGTGTTGGGCCGGGGCCGCAGGCAGCGGATGATGAAGG3′
InsertionT5R: 5′CGATGACAGTGGTCCACTGCAGGCAGCGGATGATGAAGGTGTTGGGCCGGGGCCGCTCGCAGCGGATGATGAAGGTGTTGG3′
InsertT11R: 5′CGATGACAGTGGTCCACTGCAGGCAGCGGATGATGAAGGTGTTGGGCCGGGGCCGCTCCAGGCAGCGGATGATGAAGG3′
InsertT12R: 5′CGATGACAGTGGTCCACTGCAGGCAGCGGATGATGAAGGTGTTGGGCCGGGGCCGCTCCGTCTTCATCAGCTGGCGGATGATGAAGGTGTTGGG3′
InsertT14R: 5′CGATGACAGTGGTCCACTGCAGGCAGCGGATGATGAAGGTGTTGGGCCGGGGCTGCAGGCAGCGGATGATGAAGG3′
InsertT15R: 5′CGATGACAGTGGTCCACTGCAGGCAGCGGATGATGAAGGTGTTGGGCCGGATGATGAAGGTGTTGG3′
InsertT16R: 5′CGATGACAGTGGTCCACTGCAGGCAGCGGATGATGAAGGTGTTGGGCCGGGGCCGCTGGCAGCGGATGATGAAGG3′
E17K F: 5′GCCACCATGAGCGACGTGGCTATTGTGAAGGAGGGTTGGCTGCACAAACGAGGGAAGTACATCAAGACCTGG3′
Q79K-F: 5′CATCATCCGCTGCCTGAAGTGGACCACTGTCATCG3′
Q79K-R: 5′CGATGACAGTGGTCCACTTCAGGCAGCGGATGATG3′
W80R-F: 5′CATCATCCGCTGCCTGCAGAGGACCACTGTCATCG3′
W80R-R: 5′CGATGACAGTGGTCCTCTGCAGGCAGCGGATGATG3′
Q79K–W80R-F: 5′CATCATCCGCTGCCTGAAGAGGACCACTGTCATCG3′
Q79K–W80R-R: 5′CGATGACAGTGGTCCTCTTCAGGCAGCGGATGATG3′.

### 2.5 Protein Subcellular Localization, Western Blot and Luciferase Assays

HeLa cells were transfected with constructs driving the expression of AKT1 fused to the mCherry protein. Cells were seeded, in 24-well plates containing sterile coverslips, 16 h before transfection to be confluent at the time of transfection. Cells were transfected using the calcium-phosphate method with 250 ng of plasmid per well and rinsed 24 h after transfection. At this point, cells were serum-starved or not for 24 h. Forty-eight hours after transfection, cells were washed with phosphate-buffered saline solution (PBS) and fixed for 15 min with paraformaldehyde (PFA 4%). They were washed three times in PBS and nuclei were stained with Hoechst 33342 (Invitrogen, CA, USA). Coverslips were mounted on microscope slides using the fluorescence mounting medium DakoCytomaton (DAKO, CA, USA). Cells were visualized with an epifluorescence microscope provided with an ApoTome module.

For Western-blot studies, HeLa cells were transfected with the constructs driving the expression of wild-type or mutated AKT1 forms. One day after transfection cells were rinsed and serum-starved or not for 24 h before lysis. Electrophoresis and Western-blot were performed as previously described (Georges et al., 2014). The primary antibodies used were anti-phospho-AKT (S473) (sc-7985-R, Santa Cruz Biotechnology, Dallas, TX, USA), anti-AKT (sc-8312, Santa Cruz Biotechnology, Dallas, TX, USA) and Anti-GAPDH (ABM G041, Applied Biological Materials, Inc., Richmond, BC, Canada). For pAKT1 immunohistochemistry, 4 μm-thick FFPE tumor sections were analyzed. Immunohistochemistry was carried out using the anti-phospho-AKT (S473 mentioned above) at a dilution of 1:300. Epitope unmasking was performed in a pH 6 buffer heated at 120 °C. After 20 min of incubation with the first antibody, staining was obtained using the Pure Envision dual link kit (DAKO, CA, USA) (30 min of incubation).

Dual-Luciferase Reporter Assays (Promega, Madison, WI, USA) involved the reporter promoter 4XDBE-luc which contains 4 copies of the FOXO response element (DAF-16 family member-binding element or DBE) upstream of a minimal promoter driving the expression of the firefly luciferase gene (Furuyama et al., 2000). Each experiment was performed in three replicates in 96-well plates. Cells were seeded 16 h before transfection to be at confluence at the time of transfection and transfected with 150 ng of total DNA per well (4XDBE-luc, AKT1 vector, FOXO3a (Addgen no 1787) or NLS-control vector and renilla luciferase vector) using the calcium phosphate method and rinsed 24 h after transfection. At this point, cells were serum-starved or not for 24 h. Forty-eight hours after transfection, cells were washed with PBS before lysis and luciferase measurements were performed with a TriStar LB 941 luminometer (Berthold Technologies, Bad Wildbad, Germany). To monitor transfection efficiency, a Renilla luciferase vector (pRL-RSV, Promega, Madison, WI, USA) was co-transfected. Activity is expressed as relative luciferase units (RLU, i.e. the ratio of the firefly luciferase activity over the Renilla luciferase activity). Statistical significance was estimated by Student's t-tests. Error bars represent the standard deviation between replicates.

## 3 Results

### 3.1 A Hotspot of In-frame Duplications and Point Mutations alter AKT1 in JGCTs

First of all, we performed a Sanger sequencing of the exon potentially harboring the previously reported activating mutations of *Gαs* altering protein position 201 (Kalfa et al., 2006). The absence of this mutation suggested that these tumors were good candidates to harbor driver mutations elsewhere. A survey by direct sequencing of exons 9, 18 and 20 of *PIK3CA* in 7 tumors of the cohort showed the absence of mutations suggesting that *PIK3CA* mutations were not a frequent molecular lesion in JGCTs.

**Fig. 2:**
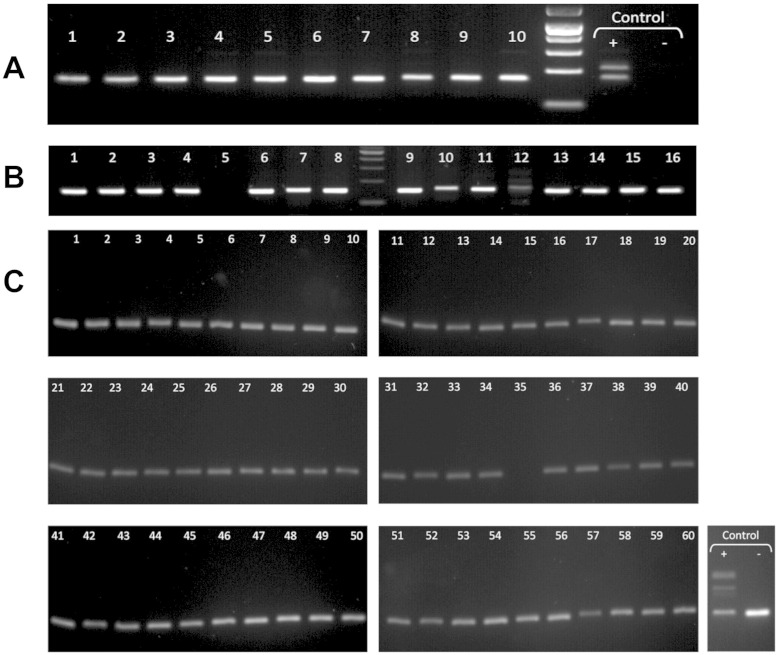
Absence of *AKT1* in-frame duplications from a series a tumor and cancer cell line genomic DNA samples. A) Exon-3 amplicons in 10 adult-type GCTs. Control + stands for exon 3 in T14 (carrying the insertion, note the bands at 154 bp and 190 bp), − denotes PCR control (without DNA). Electrophoresis migration was performed in 2% agarose gels of 24 cm to ensure allelic discrimination by size. B) Exon-3 amplicons in colorectal cancer (CRC) samples. C) Exon-3 amplicons in the 60 cell line panel of the National Cancer Institute. Control + stands for the amplicon in T13 (carrying the insertion, note the bands at 144 bp and 192 bp), − denotes PCR control (without DNA). Only one band having the wild-type length was obtained in all the samples tested thus far. Cell lines: 1: K-562; 2: MOLT-4; 3: CCRF-CEM; 4: RPMI-8226; 5: HL-60(TB); 6: SR; 7: SF-268; 8: SF-295; 9: SF-539; 10: SNB-19; 11: SNB-75; 12: U251; 13: BT-549; 14: HS 578T; 15: MCF7; 16: NCI/ADR-RES; 17: MDA-MB-231/ATCC; 18: MDA-MB-435; 19: T-47D; 20: COLO 205; 21: HCC-2998; 22: HCT-116; 23: HCT-15; 24: HT29; 25: KM12; 26: SW-620; 27: A549/ATCC; 28: EKVX; 29: HOP-62; 30: HOP-92; 31: NCI-H322M; 32: NCI-H226; 33: NCI-H23; 34: NCI-H460; 35: NCI-H522 (empty tube); 36: LOX IMVI; 37: M14; 38: MALME-3M; 39: SK-MEL-2; 40: SK-MEL-28; 41: SK-MEL-5; 42: UACC-257; 43: UACC-62; 44: IGR-OV1; 45: OVCAR-3; 46: OVCAR-4; 47: OVCAR-5; 48: OVCAR-8; 49: SK-OV-3; 50: DU-145; 51: PC-3; 52: 786–0; 53: A498; 54: ACHN; 55: CAKI-1; 56: RXF 393; 57: SN12C; 58: TK-10; 59: UO-31; 60: MDA-MB-468.

During the analysis of the *AKT1* gene, the agarose gel electrophoresis of exon-3 PCR amplicons revealed the coexistence of two or three bands in 9 out of the 16 JGCT samples (Fig. 1A). One band had the expected length and the others were longer. One sample (T1) displayed only a slow-migrating fragment. These results pointed to the existence of insertion(s) in this exon. This was confirmed through exon-3 amplification with other pairs of primers. We performed a similar analysis using the cDNAs from 4 cryopreserved tumors (T13–T16, for which high-quality mRNA was available) and again the insertions were apparent, in agreement with the results obtained with the corresponding gDNAs (Fig. 1A). An analysis of gDNA extracted from the peritumoral tissue, available only for two of the samples bearing the insertions, formally proved the somatic status of these two mutations (Fig. 1B). Sanger sequencing of the isolated DNA bands showed the presence of 10 in-frame tandem duplications, which had never been reported either in the literature or in the databases (Fig. 1C and Table 1). The slowest-migrating bands were heteroduplexes of wild-type and mutated sequences. Interestingly, all duplications but two were different, which suggests that the mutational process affecting the underlying coding region is very dynamic and attributable to DNA-polymerase errors (Viguera et al., 2001). More details on the tandem duplications (and their official names) are available in the Supplementary material. A screening by PCR of the gDNA from 10 AGCTs (Benayoun et al., 2013), 15 colorectal carcinoma samples (Benayoun et al., 2010) and 59 NCI cell lines provided no evidence for the existence of this type of insertion in such samples (Fig. 2). The latter result is in agreement with exome sequence data available for the NCI cell lines.

**Fig. 3:**
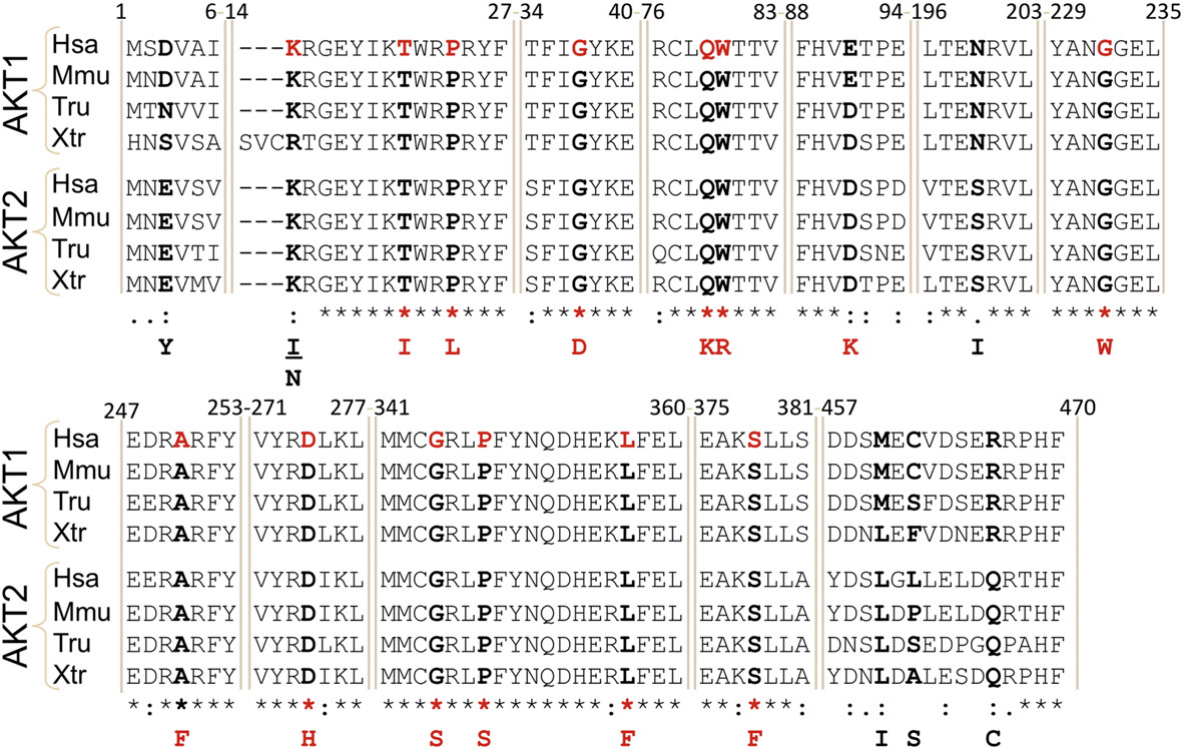
Point mutations of *AKT1* in JGCTs. Alignment of several AKT1 orthologous protein sequences from human (*Homo sapiens*, Hsa), mouse (*Mus musculus*, Mmu), puffer-fish (*Takifugu rubripes*, Tru) and the frog *Xenopus tropicalis* (Xtr) as well as the AKT2 paralogs in the same species. Note the high degree of evolutionary conservation of the mutated residues highlighted in red in the human sequence. The corresponding substitutions are displayed at the bottom of the alignment.

Given the high degree of identity between *AKT1* and *AKT2* in the region harboring the duplications, both at the DNA and protein levels, we also analyzed exon 3 of *AKT2* for the presence of duplications. No duplications could be observed. Full direct sequencing of the coding region of *AKT1* uncovered an array of point mutations altering residues highly conserved among orthologs and even between the paralogs AKT1 and AKT2 (Fig. 3 and Table 1). These mutations were identified in 10 tumors and the status of somatic mutations was confirmed for seven of them (for which peri-tumoral gDNA was available). Interestingly, several tumors without in-frame duplications carried two or more potentially damaging point mutations. Two tumors harbored homozygous/hemizygous mutations (E91N and M458I in T3 and T21I and P348S in T7). Amplicon cloning and sequencing of individual clones showed that the somatic mutations underlying the substitutions Q79K and W80R appeared on the same allele in T2. Owing to the lack of high-quality *AKT1* cDNAs for formalin-fixed paraffin-embedded (FFPE, T1–T12) samples, the allelic combinations (i.e. haplotypes) of the other co-occurring mutations could not be worked out, thus preventing their experimental exploration. In total, 14 out of 16 JGCTs harbor putative driver alterations of *AKT1*.

### 3.2 The In-frame Duplications Alter the Pleckstrin Homology Domain of AKT1 and Lead to Oncoprotein Activation

**Fig. 4:**
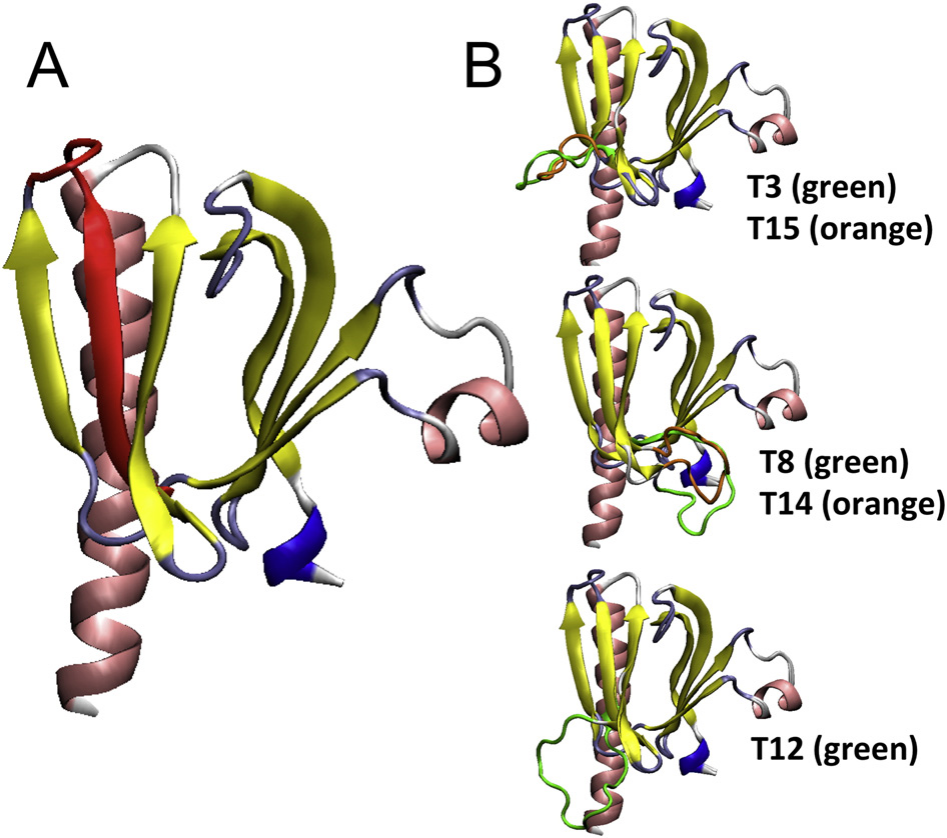
Ribbon representation of the AKT1 PHD domain and predicted effect of the duplications. A) Wild-type domain, according to the PDB Structures 1H10. The upper region mediates the interactions with the plasma membrane. The beta-strand involved in the duplications is highlighted in red. B) Predictions of the effects of several duplications on the 3D structure of the PDH domain (using Modeller Webb and Sali, 2014). We propose three main types of spatial arrangements, according to the insertions. These predictions do not take into account the presence of the rest of the protein. Structures (PDB: 3O96 and 4EJN3O964EJN) including the PHD and KD domains lack the segment linking both domains, which renders difficult the construction of realistic models. Only X-ray crystallography of both PHD and KD will allow us to work out the impact of the duplications on protein structure.

The tandem duplications described here alter the PHD of AKT1 (Gibson et al., 1994). The PHD binds to phosphatidylinositol-di/trisphosphates (PIP2 and PIP3) from the plasma membrane, which are produced by activated PI3K (Franke et al., 1997). This leads to the translocation of AKT to the plasmalemma. In such conditions, phosphoinositide-dependent kinases (among others) phosphorylate AKT1 on T308, leading to its partial activation. Full activation is achieved upon phosphorylation of S473 (Warfel et al., 2011, Mahajan and Mahajan, 2012). On structural grounds, the PHD involves two main beta sheets (Protein DataBase structures 1H10, 3O96 and 4EJN3O964EJN). The tandem duplications described here involve the 6th beta strand, according to the ribbon model displayed in Fig. 4. The co-occurring substitutions Q79K and W80R map right after the beta strand involved in the duplications. Interestingly, Q79K has already been shown to activate AKT1 probably by decreasing the interaction between the PHD and the kinase domain (Warfel et al., 2011, Yi et al., 2013) and W80 has been proposed to interact with F469 from the hydrophobic domain to keep AKT inactive (Calleja et al., 2009).

**Fig. 5:**
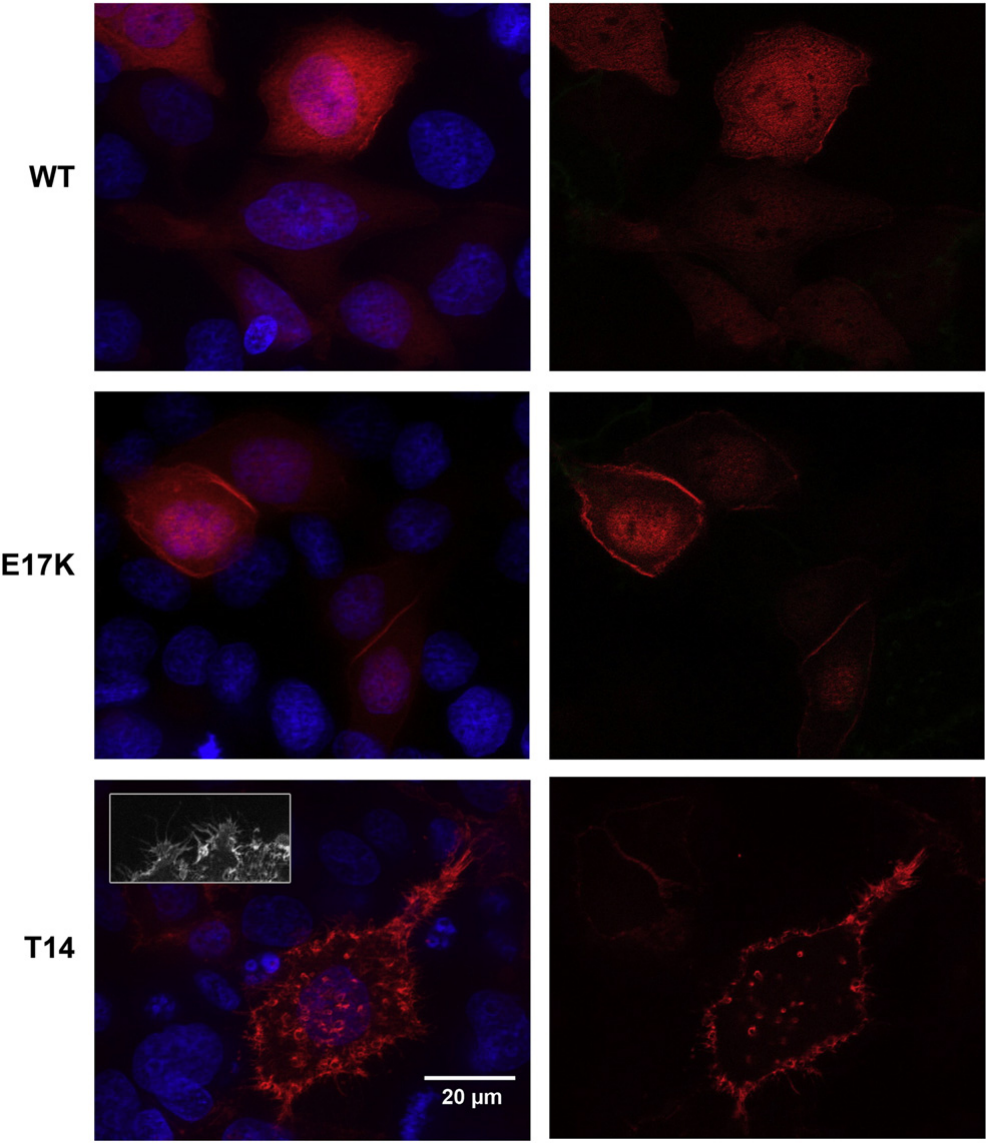
Subcellular localization of the AKT1 variants. Fluorescence microscopy of HeLa cells transfected with constructs driving the expression of different AKT1 variants fused to the mCherry fluorescent protein, in the presence of serum. Images were obtained with a Zeiss ApoTome microscope. This instrument allows the production of optical sections by using structured illumination. The left panels represent merged Z-stacks of typical cells (i.e. several images taken at different depths/focal planes within the cell). DNA was stained with Hoescht (in blue). The right panels represent optical sections, where DNA staining is not shown, to better appreciate the sub-cellular distribution of the AKT1 variants. Note the rather diffuse distribution of wild-type AKT1 and the membrane and the nuclear enrichment of E17K (activating mutation). The typical staining pattern of the mutated AKT1 carrying the duplications is strikingly different. Note the strong enrichment in the cortical sub-membrane regions. Moreover, the transfected cells displayed a profusion of filopodia-like processes (see insert). Similar results were obtained in the absence of serum. Further examples are provided in the Supplementary material.

**Fig. 6:**
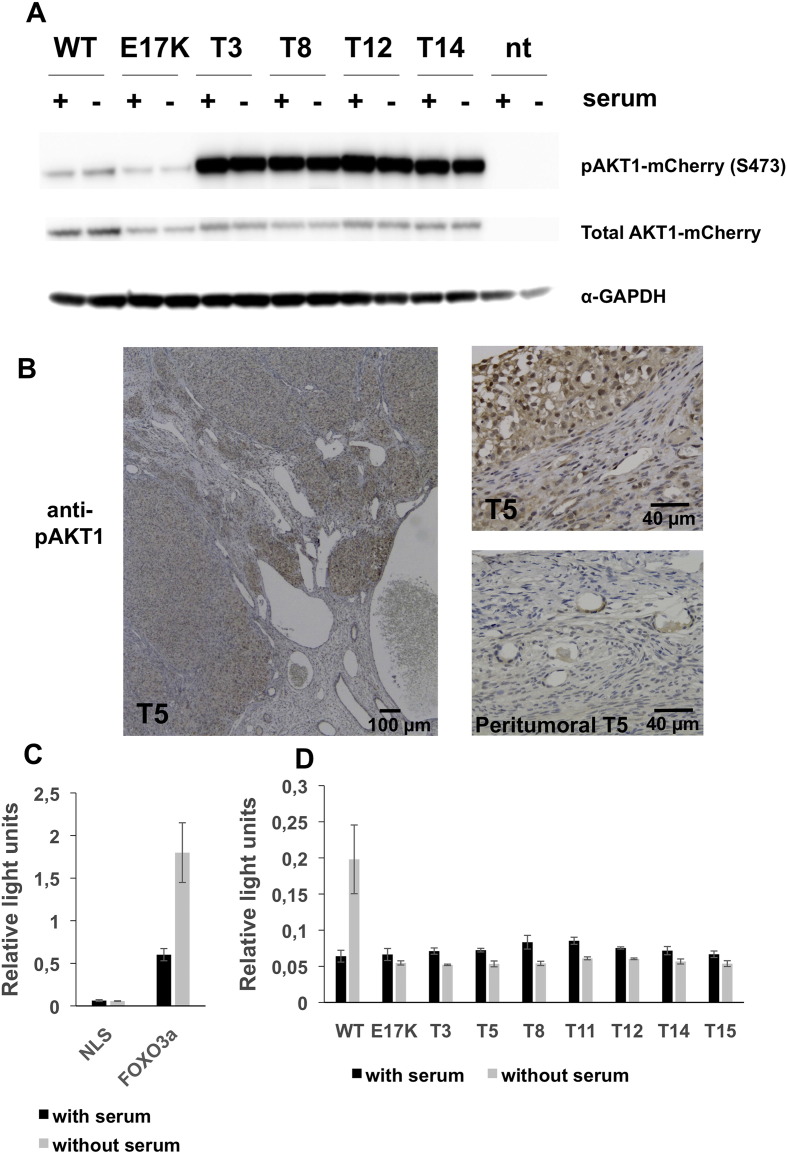
Mutated AKT1 phosphorylation and activation levels. A) Western-blot analysis of the phosphorylation levels of several AKT1 variants (wild-type/WT, E17K and 4 elongated proteins). nt: not transfected. Note that for similar amounts of total AKT1-mCherry, the mutated proteins carrying the duplications are intensely phosphorylated compared to the wild-type protein and to the E17K variant, which is our control of activating mutation. The anti-GAPDH shows that similar protein amounts were loaded in each lane, showing that cell cultures were treated and transfected very similarly. B) Anti-phospho-AKT immunostaining of a section of a tumor (T5) harboring an AKT1 tandem duplication. A section of the nearby, apparently healthy, ovarian tissue also stained with the anti-pAKT is displayed for comparison. C) Effect of the FOXO3a on the 4X-DBE-luc reporter (NLS stands for a control vector) in transfected HeLa cells in the absence or presence of serum. D) Effect of AKT1 variants on FOXO3a transactivation (note that the scale is different with respect to 6C). Here, the cells were co-transfected with 4X-DBE-luc and the various constructs driving the expression of wild-type or mutated AKT1 forms. AKT1-dependent phosphorylation of FOXO3a causes its cytoplasmic sequestration and prevents transactivation of its targets. In the presence of serum, FOXO3a was repressed by phosphorylated wild-type AKT1. In serum-starved cells, wild-type AKT1 repressed FOXO3a far less strongly. The AKT1 proteins bearing the duplications are hyperactive like the E17K mutant. In our experimental setting, this luciferase assay behaved in a rather binary way. This may explain why the impact of E17K and the duplications on AKT1 activity (measured via FOXO3a transactivation) are similar, despite the striking differences of their phosphorylation levels. Error bars represent the standard deviation of 3 replicates. The results are representative of two independent experiments. The differences between the wild-type and mutated AKT1 (in the absence of serum) estimated by a two-sided Student's t-test were all highly significant (p < 0.01).

To determine whether the duplications within the PHD induced functional alterations of AKT1, we generated constructs driving the expression of wild-type and mutated AKT1 proteins (including a version carrying the activating mutation E17K Carpten et al., 2007) fused to the mCherry fluorescent protein. We observed that the wild-type AKT1-mCherry fusion protein displayed a rather diffuse localization and that E17K was enriched at the plasma membrane but was also present in the nucleus of transfected HeLa cells, irrespective of the presence of serum. The mutated proteins carrying the duplications were almost exclusively located under the plasma membrane region of transfected cells, in the presence or absence of serum (Fig. 5 and Supplementary material). One outstanding characteristic of the cells transfected with the mutated constructs was the abundance of filopodia-like cytoplasmic processes. The subcellular localization of the mutated proteins with the duplications was strikingly different from that of E17K *AKT1* in our experimental setting. The pervasive presence at the plasmalemma of the elongated AKT1 proteins suggested the existence of a high level of activation. To test this hypothesis, we analyzed the phosphorylation status of seven AKT1 variants carrying the in-frame duplications, which is supposed to reflect protein activation (Warfel et al., 2011, Calleja et al., 2009). In agreement with our hypothesis, a Western-blot analysis using an antibody directed against phosphorylated S473 showed a dramatic difference between the wild-type and E17K variants with respect to the proteins carrying the duplications (Fig. 6A). This difference was obvious in low serum conditions as well as in the presence of serum. The presence of phosphorylated AKT1 was confirmed on histological tumor sections (Fig. 6B).

**Fig. 7:**
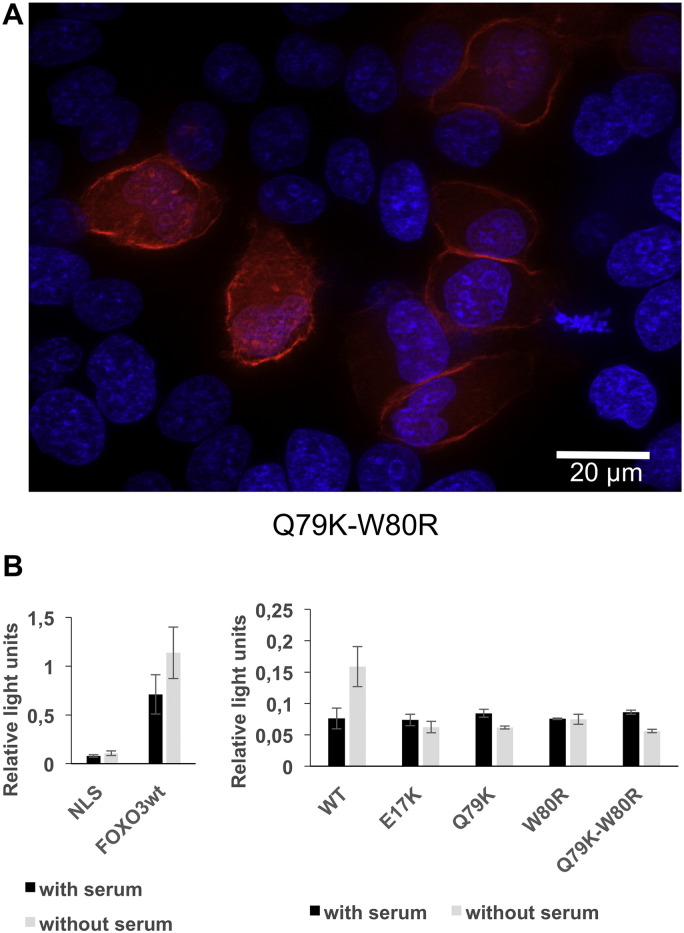
Point mutations activate AKT1. A) Fluorescence microscopy of HeLa cells transfected with constructs driving the expression of Q79K–W80R AKT1 fused to the mCherry fluorescent protein, in the same conditions as in Fig. 5. Note the strong enrichment at the cortical sub-membrane region. B) (Left panel) Effect of the FOXO3a on the 4X-DBE-luc reporter (NLS stands for a control vector) in transfected HeLa cells in the absence or presence of serum. (Right panel) Effect of two AKT1 point mutations on FOXO3a transactivation (note that the scale is different with respect to that of the left panel). The procedure was the same as in Fig. 6C, D. The AKT1 variant bearing the co-occurring mutations Q79K–W80R is hyperactive like the E17K mutant. Interestingly, each mutation alone was activating as well. Error bars represent the standard deviation of 3 replicates. The results are representative of two independent experiments. The differences between the wild-type and mutated AKT1 (in the absence of serum) estimated by a two-sided Student's t-test were all highly significant (p < 0.01). We did not attempt the analysis of the other point mutations because they co-occur with other mutations but we cannot tell apart their combinations. For instance in tumor 4, we do not know the allelic combinations encoding the mutations P24L, G232W, D274H, and S378F.

To obtain further functional insights, we studied the effect of the variants on a FOXO3a-based reporter system. Indeed, FOXO factors are negatively regulated by AKT in response to a series of growth factors and other signals. Phosphorylation of the FOXOs at three conserved sites by AKT causes their sequestration in the cytoplasm, preventing transactivation of their targets (Calnan and Brunet, 2008). Luciferase experiments were performed in HeLa cells co-transfected with the 4X-DBE-luc reporter, a FOXO3a expression vector (or a control vector) and various constructs driving the expression of wild-type or mutated AKT1 forms. Wild-type AKT1 elicited the expected response: in the presence of serum, FOXO3a was repressed by phosphorylated AKT1. In serum-starved cells, wild-type AKT1 repressed FOXO3a less strongly (Fig. 6C, D). Luciferase experiments demonstrated that the AKT1 proteins bearing the duplications were hyperactive and insensitive to serum-deprivation, much like the E17K mutant (i.e. FOXO3a was repressed irrespective of the presence or absence of serum). Of note, the mutant Q79K–W80R AKT1 also displayed strong membrane localization and hyperactivation (Fig. 7).

## 4 Discussion

The mechanisms underlying GCT formation and progression remain largely unknown and their occurrence probably results from somatic mutations. Here, we contribute to elucidate the etiology of JGCTs by showing that more than 60% of the analyzed tumors, occurring in girls under 15, bear in-frame tandem duplications in *AKT1* leading to the activation of the mutated proteins. Our functional data show that the mutated proteins display a marked membrane localization leading to AKT1 phosphorylation and to the appearance of filopodia-like processes. Of note, the tumors without *AKT1* in-frame duplications often had two or more point mutations altering highly conserved residues.

The strong enrichment at the plasma membrane of the elongated AKT1 mutants is to be correlated with that of other natural or artificial mutants such as viral-Akt, myristoylated-Akt and Akt-E40K. Interestingly, the common trait behind their efficient cell transforming capability is their enhanced localization at the plasmalemma, achieved by a PHD with increased lipotropy (E40K mutation) or by the presence of a myristoylation signal, artificially introduced or provided by the viral Gag sequence fused to Akt in v-Akt (Aoki et al., 1998). Structural studies, including crystallography, have suggested that the E17K activating mutation directly alters the lipid binding pocket of the PHD (Carpten et al., 2007). In the case of the tandem duplications identified here, it is not clear what the basis of the membrane localization and activation is. However, if we assume that the first duplicated sequence “pairs” with the 5th beta strand of the PHD (as the former emerges from the ribosome during translation) then the second copy is left unpaired and might form a protrusion. Such a structural defect might alter the interaction with the plasma membrane and/or with the kinase domain (KD). Mutations in AKT1 at the PHD–KD interface, as is the case here, that weaken their interaction have been previously reported in human cancers (Parikh et al., 2012). Such altered interactions in our mutants could explain the dramatic tropism for the plasmalemma and the concomitant increase of phosphorylation. A similar explanation would hold for the co-occurring mutations Q79K–W80R. However, further studies are required to completely elucidate the underlying mechanism(s).

Although JGCTs are of good prognosis, it would be interesting to study the properties of the filopodia-like processes induced by the mutated AKT1 carrying the duplications because of the known roles of filopodia/invadopodia in sensing, migration and intercellular interactions (Mattila and Lappalainen, 2008). These processes are similar to those observed upon eEF1A2 over-expression in BT549 human breast cancer cells and non-transformed Rat2 cells. Interestingly, eEF1A2 expression in BT549 cells stimulates filopodia formation, cell migration and invasion in a PI3K- and Akt-dependent manner (Amiri et al., 2007). Along the same vein, squamous cell carcinoma lines engineered to express constitutively active AKT display down-regulation of E-cadherin, reduced cell–cell adhesion and increased motility in vivo (Grille et al., 2003).

One interesting question is raised by the apparent specificity of the AKT1 insertions. Several hypotheses can explain this fact. First, the tandem duplications only appear in the ovary because the underlying mutational process is favored in granulosa cells for as yet unknown reasons. Second, the selective advantage of tumor cells may involve interactions of the mutated AKT1 with ovarian-specific (signaling or interacting) partners. Finally, we cannot exclude that granulosa cells may need a strong level of AKT1 activation to become transformed, which would only be achieved with the observed duplications or with several AKT1 mutations per cell, such as Q79K and W80R. The fact that most of the JGCTs harbor *AKT1* mutations and the involvement of the PI3K–AKT pathway in regulating cellular proliferation and survival suggests that the molecular lesions reported here are driver events. Irrespective of the precise role of AKT1 in the origin and/or progression of the JGCTs, which is yet to be studied, the uncovering of the *AKT1* insertions will facilitate the molecular diagnostics of JGCTs. Our findings open also targeted therapeutic perspectives because inhibitors of the PI3K–AKT–mTOR pathway(s) are being tested in clinical trials (Don and Zheng, 2011).

## Funding

This work was supported by GEFLUC (Groupement des Entreprises Françaises dans la Lutte contre le Cancer), the Centre National de la Recherche Scientifique (CNRS), the Université Paris Diderot and the Agence Nationale pour la Recherche (Projet ICEBERG).

## Author Contributions

LB, ALT, AA and RAV conceived and designed the study. LB, ALT, AA, LG, BL, DF and RAV developed the methodology. LB, ALT, AA, LG, BL and RAV acquired data. LB, ALT, AA, SS, DF, CS, NK, LG and RAV analyzed and/or interpreted data. All authors wrote and provided final approval of the manuscript.

## Declaration of Interests

The authors declare no competing interests.

## Ethics Committee Approval

This project was approved by the ethics committees of the tumor repositories *Tumorothèque Necker-Enfants Malades* and *Tumorothèque Montpellier*.

## Role of the Funding Source

The funding sources had no role in study design, data collection, data analysis, data interpretation, or writing of the manuscript. The corresponding author had full access to all data and had the final responsibility for the decision to submit for publication.

## Figures and Tables

**Table 1:**
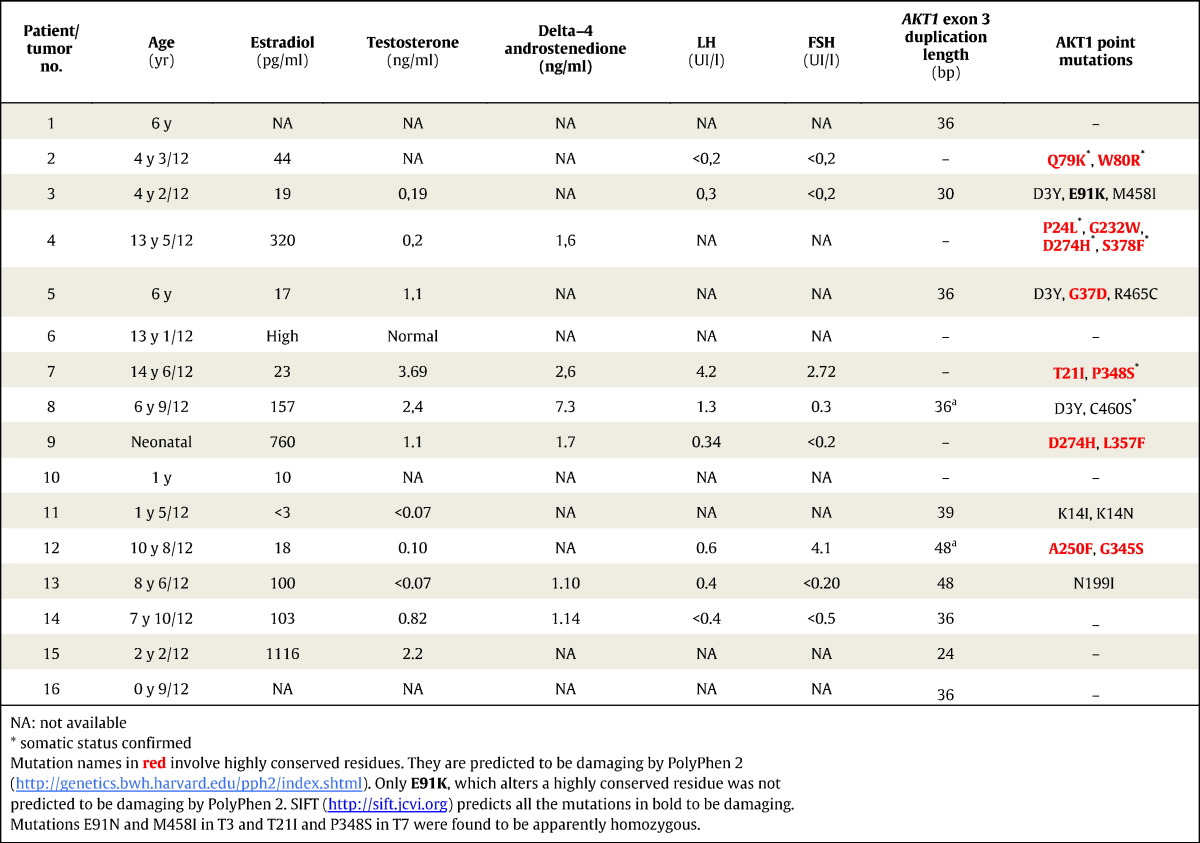
Clinical features of patients with JGCTs.

## Appendix A Supplementary Data

Details of the tandem duplications and their impact on protein localization.
