# Supplementary material for: A Hot-spot of In-frame Duplications Activates the Oncoprotein AKT1 in Juvenile Granulosa Cell Tumors
Source: eBioMedicine. 2015 Mar 6;2(5):421–31. doi: 10.1016/j.ebiom.2015.03.002 (PMC4485906; doi:10.1016/j.ebiom.2015.03.002)
Supplement: Supplementary file 1 — Details of the tandem duplications and their impact on protein localization. [file mmc1.pdf]

Supplementary Results

The figures below display the electropherograms of the duplications, pair-wise alignments of the wild-type and mutated AKT1 sequences and official mutation names.

Tumor 1

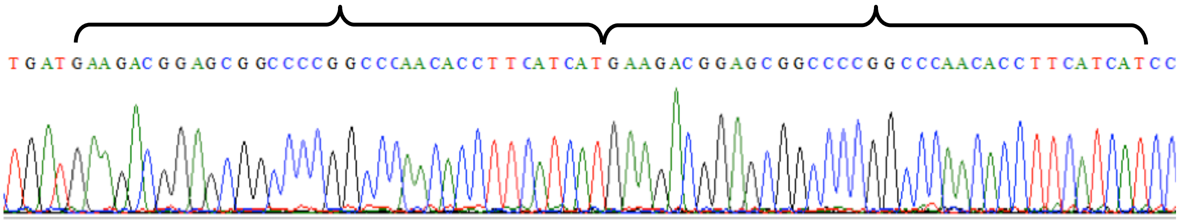

```
WT                RPQDVDQREAPLNNFSVAQCQLMKTERPRPNTFI------------IRCLQWTTVIERTF 48
T1                RPQDVDQREAPLNNFSVAQCQLMKTERPRPNTFIMKTERPRPNTFIIRCLQWTTVIERTF 60
                  **********************************            **************
```

Official Mutation name. NM_001014431(AKT1_v001):c.189_224dup, p.(Met63_Ile74dup)

Tumor 3

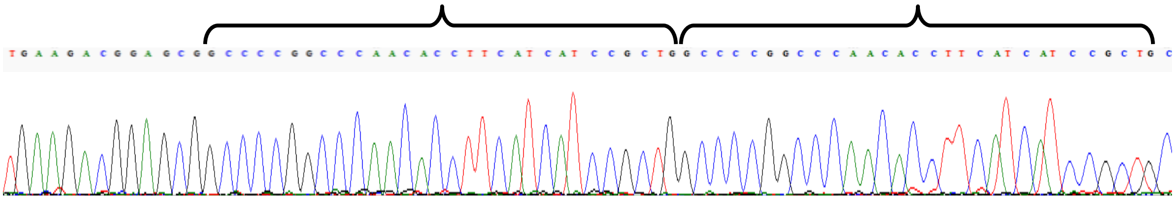

```
WT                RPQDVDQREAPLNNFSVAQCQLMKTERPRPNTFIIR----------CLQWTTVIERTFHV
T3                RPQDVDQREAPLNNFSVAQCQLMKTERPRPNTFIIRWPRPNTFIIRCLQWTTVIERTFHV
                  ************************************          **************
```

Official Mutation name. NM_001014431(AKT1_v001):c.201_230dup,
p.(Arg76_Cys77insTrpProArgProAsnThrPheIleIleArg)

Tumor 5

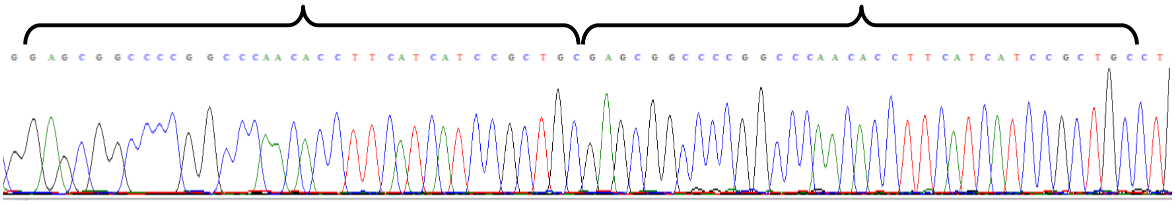

```
WT                RPQDVDQREAPLNNFSVAQCQLMKTERPRPNTFIIRC------------LQWTTVIERTF 48
T5                RPQDVDQREAPLNNFSVAQCQLMKTERPRPNTFIIRCERPRPNTFIIRCLQWTTVIERTF 60
                  *************************************            ***********
```

Official Mutation name. NM_001014431(AKT1_v001):c.196_231dup, p.(Glu66_Cys77dup)

Tumor 8

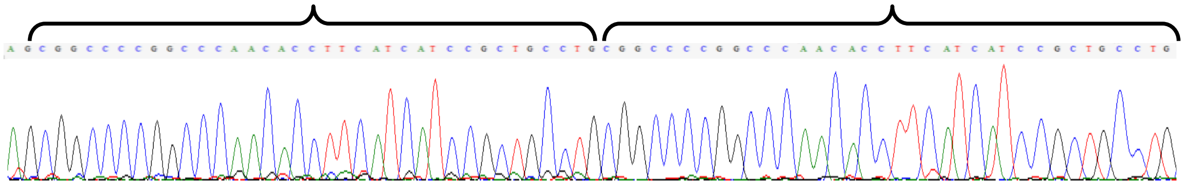

```
WT      RPQDVDQREAPLNNFSVAQCQLMKTERPRPNTFIIRCL------------QWTTVIERTF 48
T8      RPQDVDQREAPLNNFSVAQCQLMKTERPRPNTFIIRCLRPRPNTFIIRCLQWTTVIERTF 60
        **************************************            **********
```

Official Mutation name. NM_001014431(AKT1_v001):c.200_235dup, p.(Arg67_Leu78dup)

Tumor 11

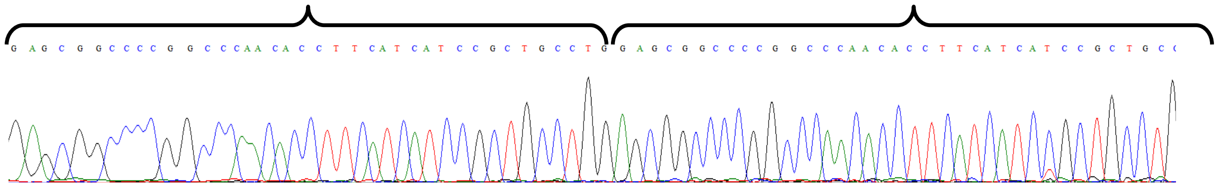

```
WT      RPQDVDQREAPLNNFSVAQCQLMKTERPRPNTFIIRCL-------------QWTTVIERT 47
T11     RPQDVDQREAPLNNFSVAQCQLMKTERPRPNTFIIRCLERPRPNTFIIRCLQWTTVIERT 60
        **************************************             *********
```

Official Mutation name. NM_001014431(AKT1_v001):c.196_234dup, p.(Glu66_Leu78dup)

Tumor 12

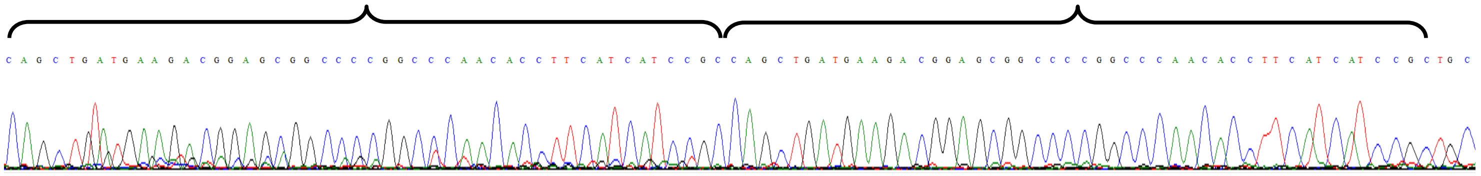

```
WT      RPQDVDQREAPLNNFSVAQCQLMKTERPRPNTFIIR----------------CLQWTTVI 44
T12     RPQDVDQREAPLNNFSVAQCQLMKTERPRPNTFIIRQLMKTERPRPNTFIIRCLQWTTVI 60
        ************************************                ********
```

Official Mutation name. NM_001014431(AKT1_v001):c.181_228dup, p.(Gln61_Arg76dup)

Tumor 13

```
WT                RPQDVDQREAPLNNFSVAQCQLMKTERPRPNTFIIR----------------CLQWTTVI 44
T13               RPQDVDQREAPLNNFSVAQCQLMKTERPRPNTFIIRQLMKTERPRPNTFIIRCLQWTTVI 60
                  ************************************                ********
```

Official Mutation name. NM_001014431(AKT1_v001):c.181_228dup, p.(Gln61_Arg76dup)

Tumor 14

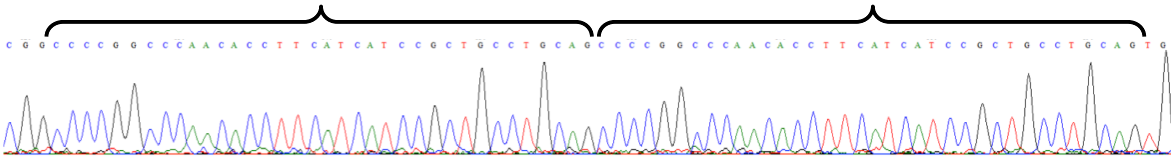

```
WT                RPQDVDQREAPLNNFSVAQCQLMKTERPRPNTFIIRCLQ------------WTTVIERTF 48
T14               RPQDVDQREAPLNNFSVAQCQLMKTERPRPNTFIIRCLQPRPNTFIIRCLQWTTVIERTF 60
                  ***************************************            *********
```

Official Mutation name. NM_001014431(AKT1_v001):c.202_237dup, p.(Pro68_Gln79dup)

Tumor 15

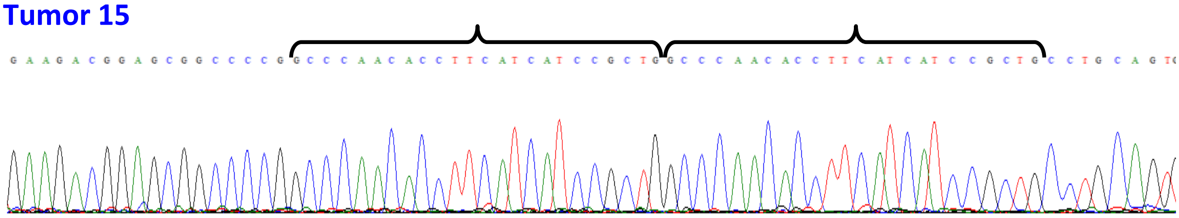

Alignment of the AKT1 wild-type and mutated sequences.

```
WT                RPQDVDQREAPLNNFSVAQCQLMKTERPRPNTFIIR--------CLQWTTVIERTFHVET 52
T15               RPQDVDQREAPLNNFSVAQCQLMKTERPRPNTFIIRWPNTFIIRCLQWTTVIERTFHVET 60
                  *****************************        ***********************
```

Official Mutation name. NM_001014431(AKT1_v001):c.207_230dup, p.(Arg76_Cys77insTrpProAsnThrPheIleIleArg)

Tumor 16

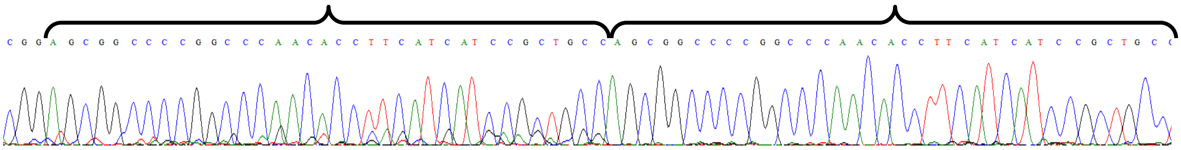

Alignment of the AKT1 wild-type and mutated sequences.

```
WT                RPQDVDQREAPLNNFSVAQCQLMKTERPRPNTFIIRC------------LQWTTVIERTF 48
T16               RPQDVDQREAPLNNFSVAQCQLMKTERPRPNTFIIRCQRPRPNTFIIRCLQWTTVIERTF 60
                  *************************************            ***********
```

Official Mutation name. NM_001014431(AKT1_v001):c.197_232dup, p.(Cys77_Leu78insGlnArgProArgProAsnThrPheIleIleArgCys)

The figures below display fluorescence microscopy images of HeLa cells transfected with constructs driving the expression of different AKT1 variants fused to the mCherry fluorescent protein, in the presence of serum. Images were obtained with a Zeiss ApoTome microscope. Scale bars in the figures stand for 20 μm.

Tumor 3. Mutation p.(Arg76_Cys77insTrpProArgProAsnThrPheIleIleArg)

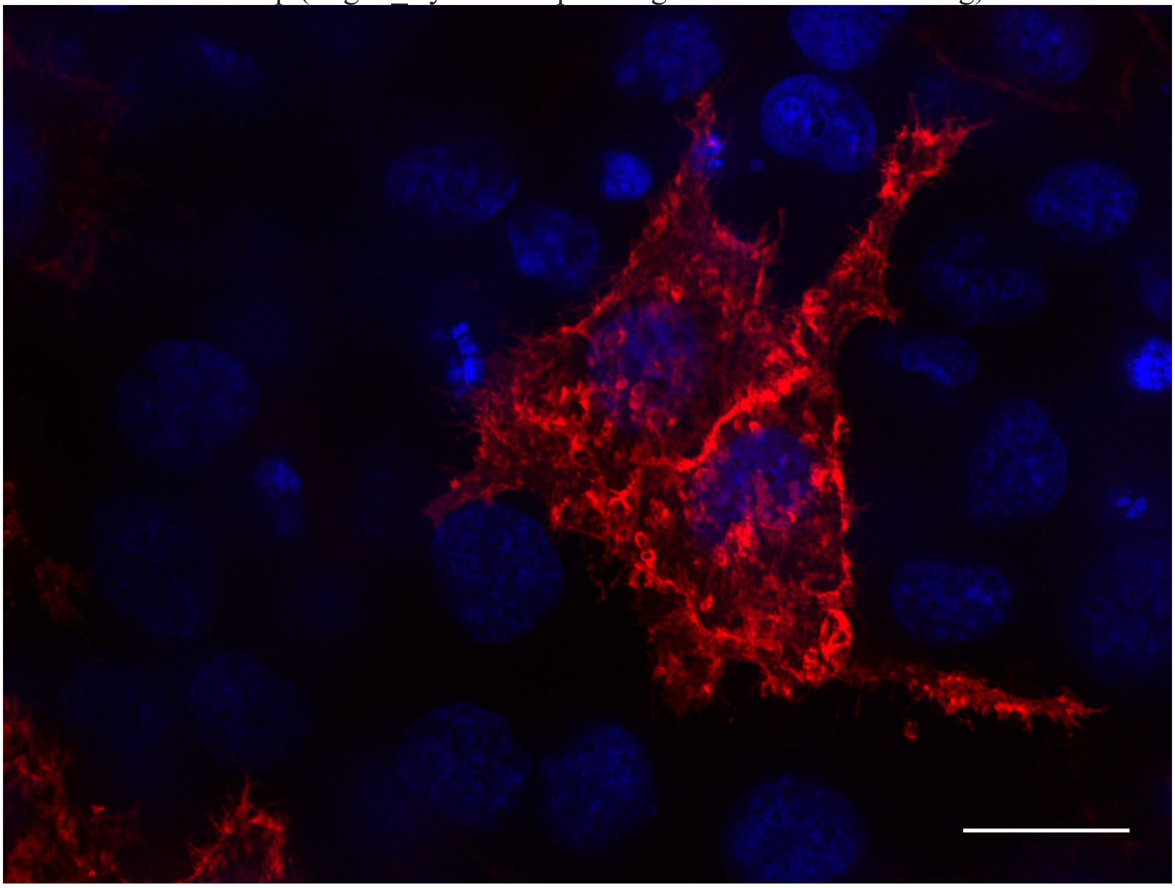

Tumor 5. Mutation p.(Glu66_Cys77dup)

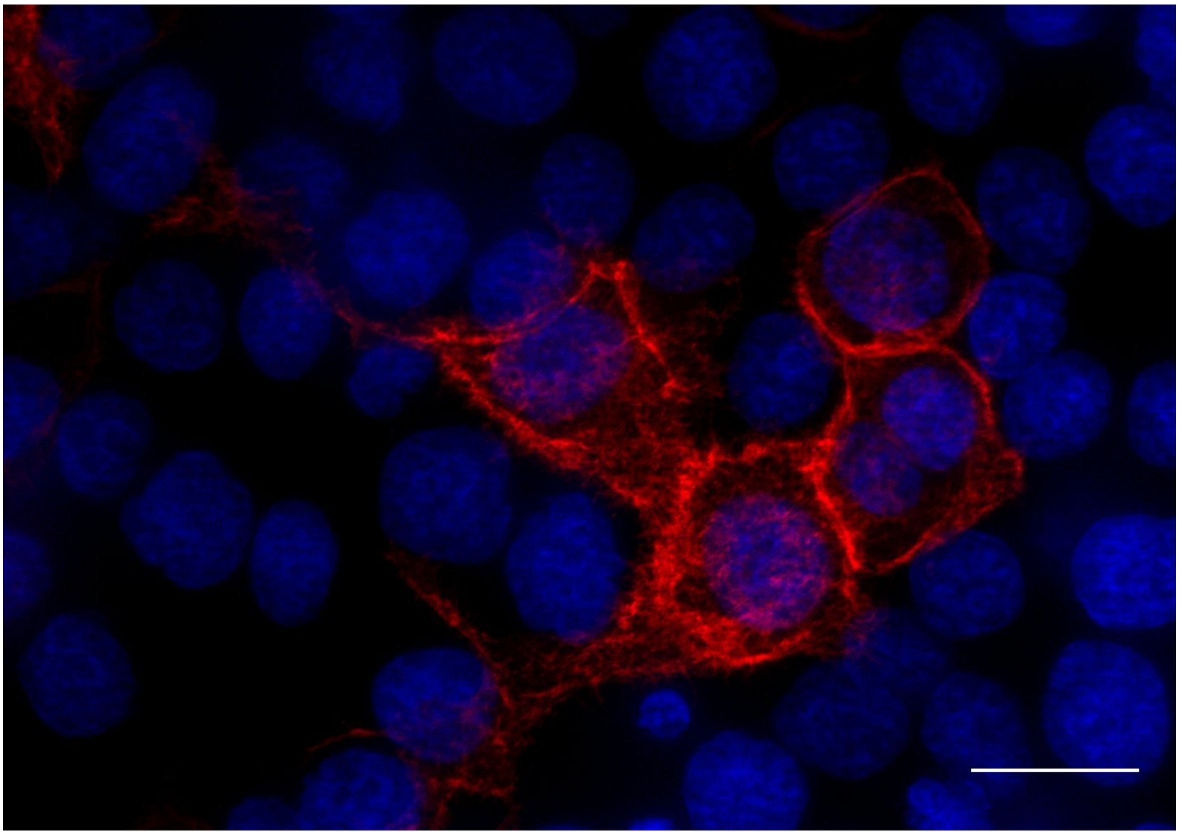

Tumor 8. **Mutation** p.(Arg67_Leu78dup)

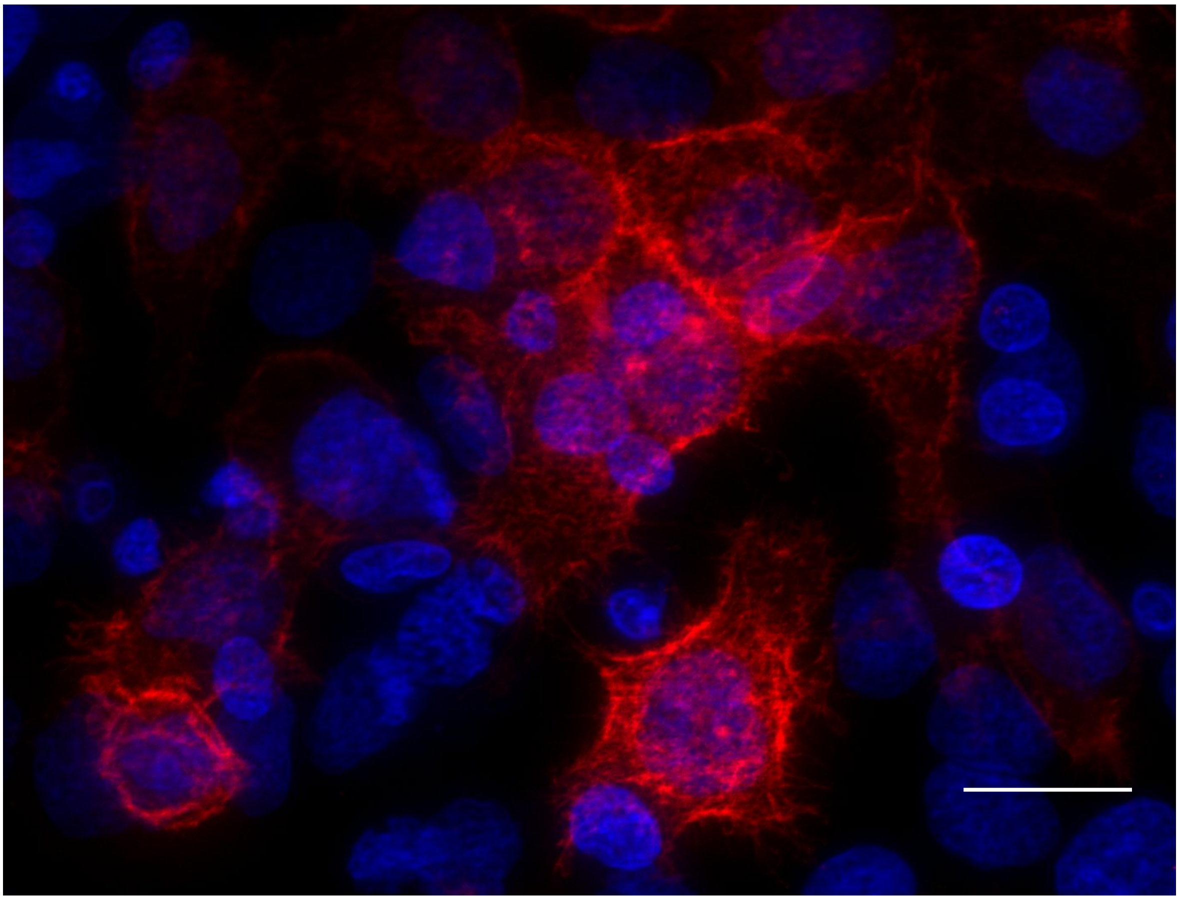

Tumor 11. **Mutation** p.(Glu66_Leu78dup)

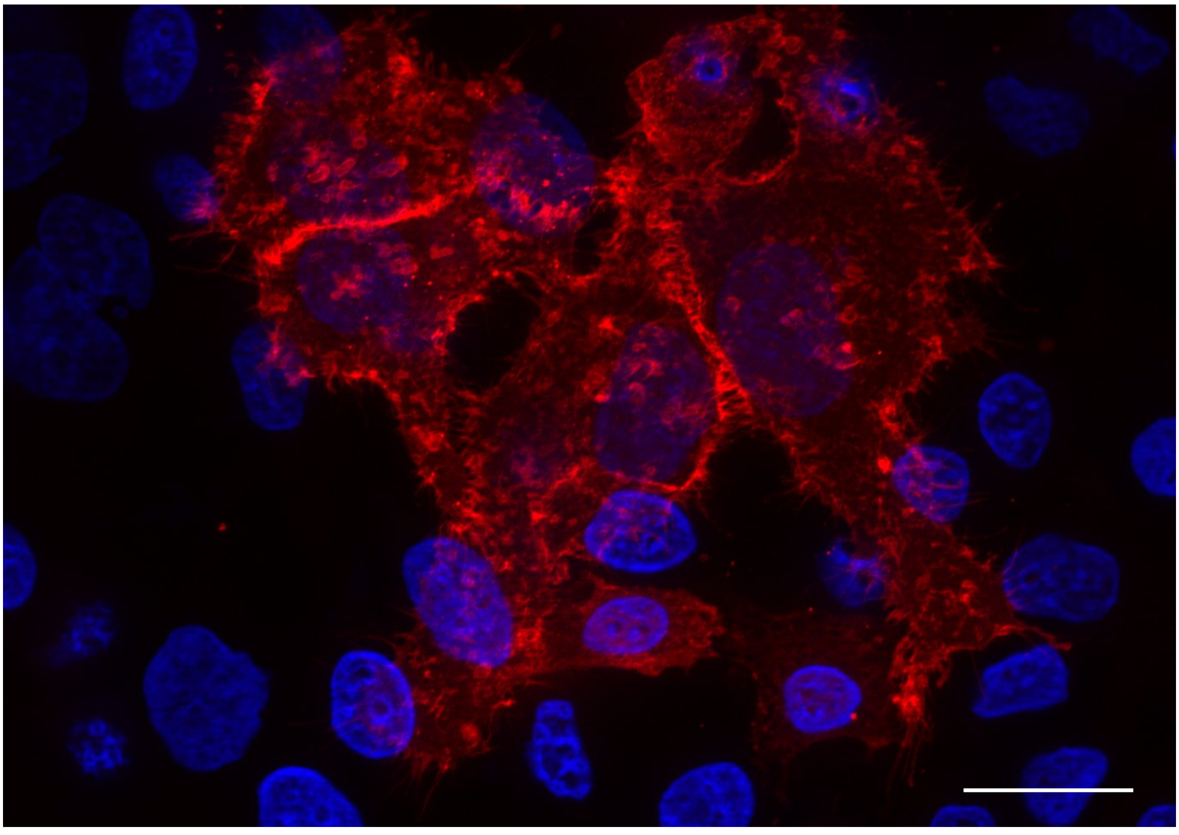

Tumor 12. **Mutation** p.(Gln61_Arg76dup)

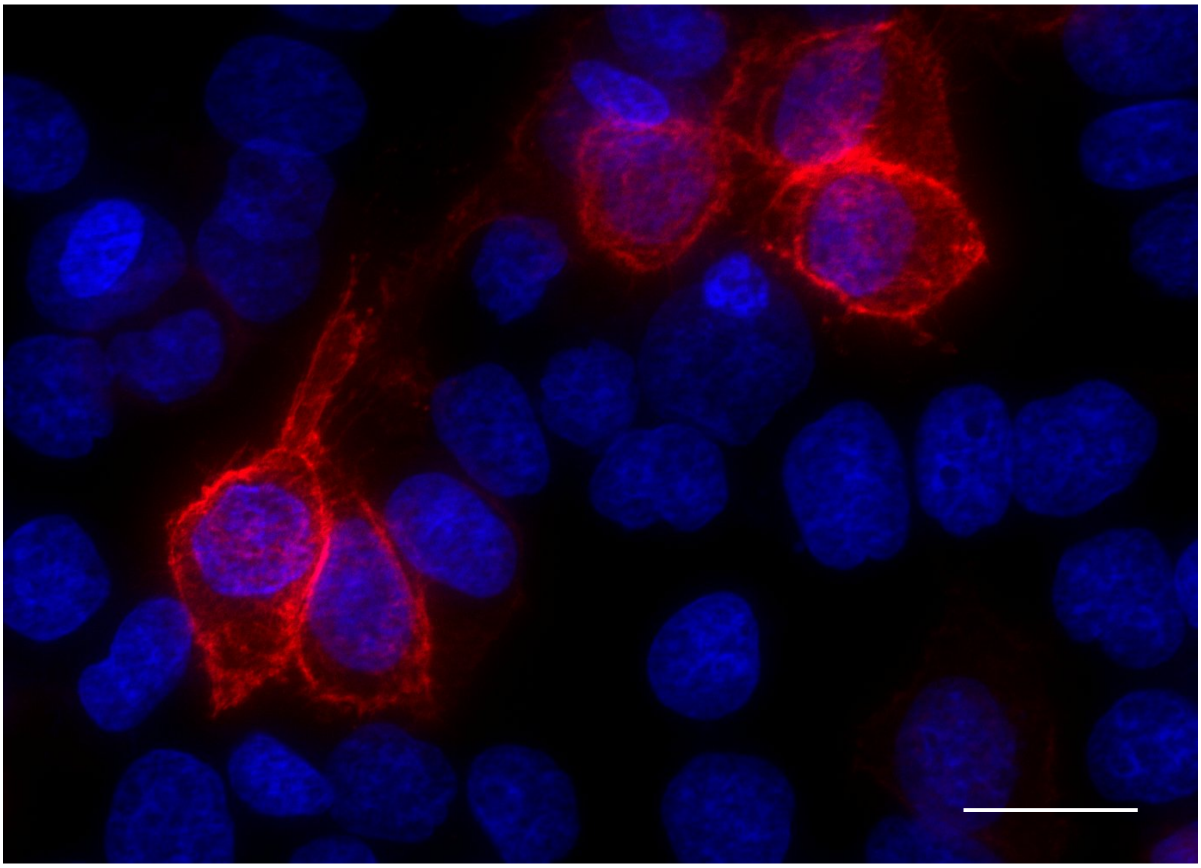

Tumor 16. **Mutation** p.(Cys77_Leu78insGlnArgProArgProAsnThrPheIleIleArgCys)

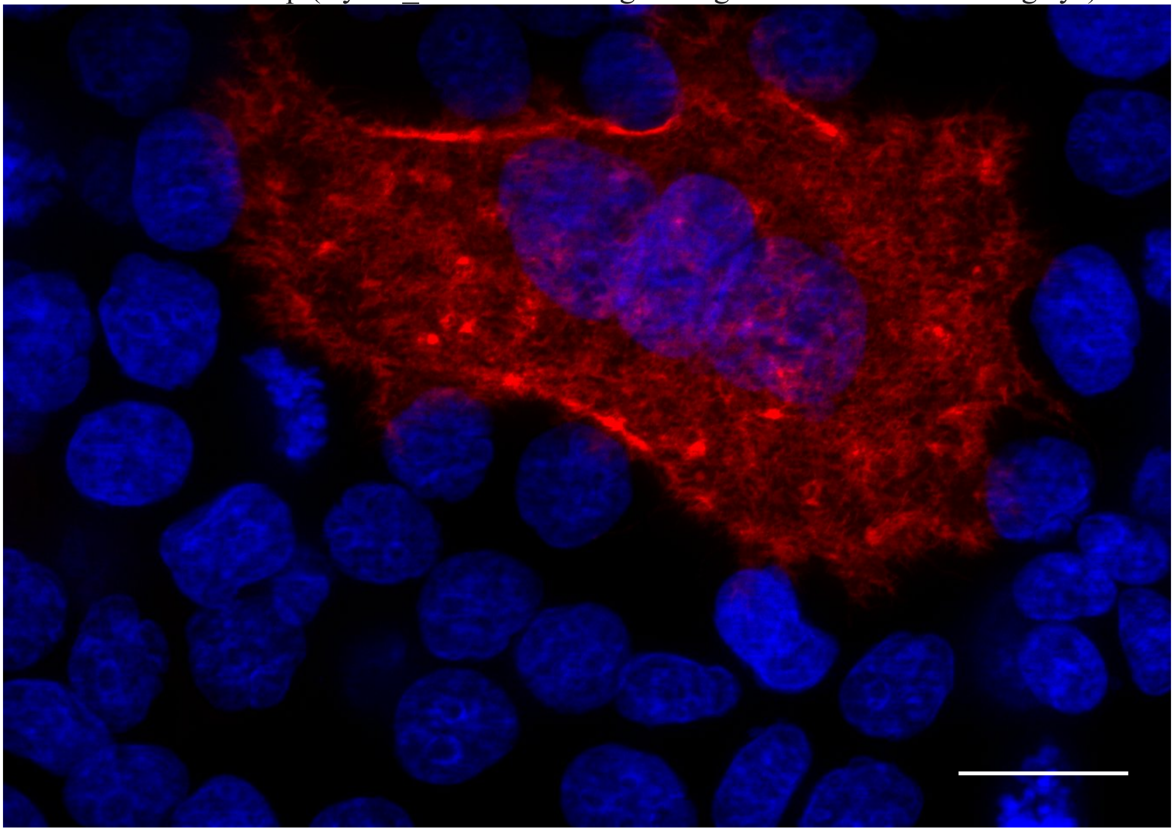

Point mutation analysis of AKT1

Table S1 : Summary of the point mutation analysis

Name (protein)	Name (nucleic acid)	Substitu-tion	Affected tumor	Affected exon	Homozygous (a/a) Heterozygous (a/b)	Absence in somatic tissu
**p.D3Y**	c.7g>T	gac>Tac	5, 8	1	a/b	NA
**p.K14I**<br>**p.K14N**	c.40a>T<br>c.41a>T	aaa>aTa<br>aaa>aaT	11	1	a/b	NA
**p.T21I**	c.62c>T	acc>aTc	7	2	a/a	NA
**p.P24L**	c.71c>T	cca>cTa	4	2	a/b	Yes
**p.G37D**	c.110g>A	ggc>gAc	5	2	a/b	NA
**p.Q79K**	c.235c>A	cag>Aag	2	3	a/b	Yes
**p.W80R**	c.238t>A	tgg>Agg	2	3	a/b	Yes
**p.E91K**	c.271g>A	gag>Aag	3	3	a/a	NA
**p.N199I**	c.537a>T	aac>aTc	13	6	a/b	NA
**p.G232W**	c.695g>T	ggg>Tgg	4	7	a/b	NA
**p.A250F**	c.749c>T	gcc>gTc	12	8	a/b	NA
**p.D274H**	c.820g>C	gac>Cac	4, 9	8	a/b	Yes (T4)
**p.G345S**	c.1033g>A	ggt>Agt	12	10	a/b	NA
**p.P348S**	c.1042c>T	ccc>Tcc	7	10	a/a	NA
**p.L357F**	c.1069c>T	ctt>Ttt	9	10	a/b	NA
**p.S378F**	c.1133c>T	tcc>tTc	4	10	a/b	Yes
**p.M458I**	c.1374g>A	atg>atA	3	13	a/a	NA
**p.C460S**	c.1378t>A	tgt>Agt	8	13	a/b	Yes
**p.R465C**	c.1393c>T	cgc>Tgc	5	13	a/b	NA

NA : not available
